# Early or Late Feeding after ICU Admission?

**DOI:** 10.3390/nu9121278

**Published:** 2017-11-23

**Authors:** Annika Reintam Blaser, Mette M. Berger

**Affiliations:** 1 Department of Anaesthesiology and Intensive Care, University of Tartu, 51014 Tartu, Estonia; 2 Department of Intensive Care Medicine, Lucerne Cantonal Hospital, 6000 Lucerne, Switzerland; 3 Service of Intensive Care and Burns, Lausanne University Hospital (CHUV), 1011 Lausanne, Switzerland; Mette.Berger@chuv.ch

**Keywords:** enteral nutrition, early feeding, parenteral nutrition, starvation, critical illness

## Abstract

The feeding of critically ill patients has recently become a controversial issue, as several studies have provided unexpected and contradictory results. Earlier beliefs regarding energy requirements in critical illness—especially during the initial phase—have been challenged. In the current review, we summarize existing evidence about fasting and the impact of early vs. late feeding on the sick organism’s responses. The most important points are the non-nutritional advantages of using the intestine, and recognition that early endogenous energy production as an important player in the response must be integrated in the nutrient prescription. There is as of yet no bedside tool to monitor dynamics in metabolism and the magnitude of the endogenous energy production. Hence, an early “full-feeding strategy” exposes patients to involuntary overfeeding, due to the absence of an objective measure enabling the adjustment of the nutritional therapy. Suggestions for future research and clinical practice are proposed.

## 1. Introduction

Over the last decades, nutritional interventions carried out in critically ill patients were characterized by different timings, strategies, and terminologies. Recent randomised trials have brought some new insights in this topic, challenging several earlier beliefs. Early full nutrition—which is achieved more easily with parenteral nutrition (PN)—is associated with negative clinical outcomes, whereas the route itself may be irrelevant [1]. In the case of enteral nutrition (EN), full feeding may actually invalidate its beneficial effects, as shown by two randomised trials—the CALORIES trial [2] and the recent EAT-ICU trial [3]. However, “early nutrition” and “full feeding” have not been uniformly defined. Confusion also results from the enrolment of very heterogeneous patient populations presenting a large scale of severity and diagnostic categories.

After clarifying some important definitions (Table 1), the aim of this narrative review is to summarize the physiological and clinical evidence regarding the consequences of early vs. late feeding, and give suggestions on how to transfer this knowledge to practice.

## 2. Metabolic Response to Fasting

**Figure 1:**
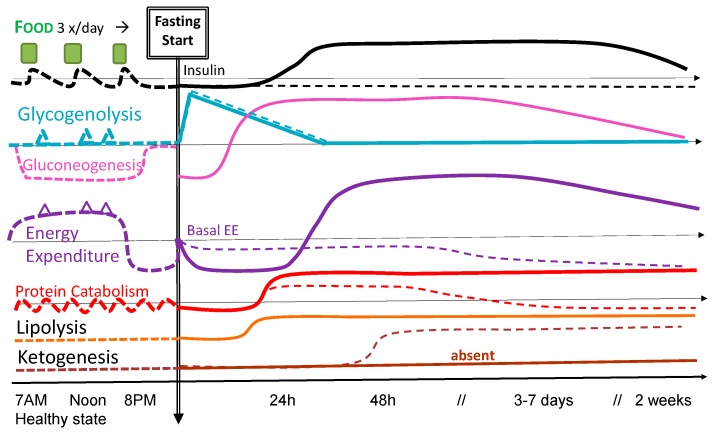
Conceptual representation of the metabolic consequences of fasting (complete interruption of feeding) in healthy subjects (dotted lines) and critically ill patients (full lines) on the different pathways. Thin horizontal lines represent the normal 100% value. Triangles = physical activity in healthy subjects. EE, energy expenditure.

Considering the question of early or late feeding implies that there is a variable period of fasting. Significant misunderstanding regarding consequences may be formed, should the metabolic tolerance and patterns of responses similar in healthy [4] and sick patients be considered: Figure 1 shows some of these differences. The neuroendocrine response that characterizes acute illness deeply modifies metabolism, and tolerance to fasting is therefore altered [6]. However, the exact mechanisms are still poorly understood, and have only partially been investigated. Ideally, precise monitoring of metabolic responses should guide nutrition therapy, including the decision of whether or not to initiate early feeding. Unfortunately, such in-depth monitoring is currently not available.

In healthy subjects, fasting is considered to have potential beneficial effects, including spiritual benefits [4,7]: multiple patterns of fasting have been described, such as intermittent fasting [5]. During chemotherapy, some studies show that short fasting may be beneficial [8]. In acute severe illness, tolerable duration of fasting and eventual beneficial effects are not clear, whereas the multi-faceted nature of critical illness makes the likelihood of a “one-size-fits-all” correct answer unlikely.

Fasting is the complete interruption of feeding for a variable duration, while nutrient restriction can be defined as the reduction of particular or total nutrient intake without causing malnutrition [5]. In critically ill patients, the situation is rarely an absolute fasting due to the delivery of glucose and fat from fluid (glucose and glucosaline) or drugs—the so called non-nutritional energy sources: these intakes vary between 50 and sometimes 400 kcal/day, and are by definition unbalanced from a nutritional point of view [9]. While all organs need continuous oxygen and energy supply, some of them (e.g., the brain) have greater needs than others [10]. Both healthy subjects and sick patients require a continuous glucose fuelling of the central nervous system and blood cells, and hence need a constant plasma glucose concentration. The integrated adaptation mechanisms to prevent energy insufficiency activate different pathways. In health, fasting can be described as a three-step process [11]: (1) an *overnight fasting* (defined as 14-h fasting or post-absorptive state); (2) a *short term fasting* up to 85 hours of starvation in which progressive alterations in lipid and glucose metabolism occur; (3) *prolonged fasting beyond 3 days* that causes protein catabolism.

In healthy subjects, plasma glucose levels decrease during fasting due to the slowly decreasing endogenous glucose production (EGP) from glycolysis and gluconeogenesis (dotted lines in Figure 1). In resting circumstances, glycogen stores will be reduced to a minimum after approximately 40 h of fasting with a reduced liver size [12], after which EGP primarily relies on gluconeogenesis. The adaptation is maximal within about 3 days. Thereafter, several important changes in energy metabolism occur with persistent hypoinsulinemia, decreased glucose oxidation, increased fatty acid oxidation and lipolysis, increasing ketogenesis, as well as decreased insulin-mediated glucose uptake, glycogenolysis, gluconeogenesis, and proteolysis. Plasma ketone bodies become an important source of energy, covering about a third of the energy needs of the brain and of the heart. In healthy subjects, the use of substrates is largely determined by the composition of the diet and the time elapsed since the last meal. Short-term fasting induces a beneficial insulin resistance in healthy subjects, helping to maintain blood glucose levels and thereby improving survival capacity [13]. When *refeeding* finally occurs, fat is processed as in the normal fed state, but the liver at first remains in a gluconeogenic mode. As the blood-glucose levels continue to rise, the liver completes the replenishment of its glycogen stores and begins to process the remaining excess glucose for fatty acid synthesis.

In sick patients, the response is modulated by the intensity of the inflammatory response (absent in healthy subjects) and the intensity of the energy expenditure (EE) changes, causing persistent protein catabolism [6]. The magnitude of EE changes will depend on the stage and type of disease, and on the timing point into disease [14]: EE is generally depressed during states of shock and the early stages of severe illness [15] and increases variably thereafter, depending on the intensity of the inflammatory response and the amount of tissue to repair. While considered the gold standard, indirect calorimetry is yet not widely available, which complicates the evaluation of the optimal energy targets and the interpretation of the results. Making indirect calorimetry more available, and evaluating whether and at which time point the measured EE really becomes the target to be prescribed for feeding, is an important issue to consider [14,16].

The neuroendocrine response to injury is a complex response that primarily stimulates protein catabolism [17], and is aggravated by being bed-ridden and immobilized [18]. The immune system and injured tissues become major glucose consumers as insulin resistance develops, reorganizing the distribution of the substrates. Glucose becomes the preferential substrate, with an intense stimulation of the endogenous glucose production (EGP) that may reach 1200 kcal/day [19]. EGP will remain elevated for prolonged periods, and is not directly suppressed by feeding. Ketogenesis is nearly abolished, rendering the body dependent on EGP [19] to fuel the leukocytes and the brain. The magnitude and duration of the described metabolic response is very variable.

A major factor limiting optimal management is the inability to measure the dynamics of the glucose, protein, and lipid turnover at the bedside of an individual patient. Having such monitoring tools would enable targeted nutrition and metabolic therapy to control catabolism and support recovery.

## 3. Gastrointestinal Dysfunction

While all guidelines favour EN, they do not address the important dysfunctions that affect the gastrointestinal (GI) tract in response to critical illness, and make the use of this route problematic in many patients [20,21]. Multiple factors reduce the efficacy of EN: hypoperfusion, fluid resuscitation, analgo-sedation, vasoactive agents, etc. all contribute to development of GI motility disorders. Large fluid resuscitation volumes result in a generalized intestinal edema [22], leading to impaired absorption, motility, and lymph flow. Transit is frequently slowed, with delayed passage of stools (constipation) in 70% of patients [23], requiring laxative therapy with resultant diarrhoea. When diarrhoea exceeds 350 g/day, malabsorption of nutrients is probable [24]. Intestinal absorption is indeed variable and unpredictable, being influenced by several factors, such as motility, enterocyte function, splanchnic perfusion, properties of the food, etc. When motility is decreased, the absorption is unreliable [25]. Although EN is the best stimulant of GI motility, persisting feeding intolerance (therapy-refractive gastroparesis or bowel paralysis) may preclude its administration. When feeding is delayed, glucose absorption decreases consistently with the reduction in mucosal integrity after nutrient deprivation, as evident in animal models [26].

The advantages of EN include the maintenance of gut integrity and function, and modulation of the stress and immune responses [27]. However, the eventual alterations in absorption are rarely considered and are difficult to monitor, yet may lead to relevant deficiencies of both macro- and micronutrients. The impact of proton pump inhibitors on the digestibility of nutrients, the impact of diet composition on absorption, and the secretion of digestive enzymes during continuous EN are questions that are poorly studied and understood [28].

It is not clear if the systematic and early use of prokinetic agents to stimulate GI motility is beneficial or harmful [29]. In an observational study [30], after implementation of the PepUP protocol, surgical patients received a smaller proportion of prescribed energy (43% vs. 61%, *p* = 0.004) and protein (38% vs. 57%, *p* = 0.002) compared to medical patients. Whether an improper protocol implementation (as suggested by the authors) may explain this finding or if the aggressive use of prokinetics played a role remains to be demonstrated.

## 4. What Do We Know about Early Nutrition?

When feeding resumes after fasting, patients are exposed to the risk of refeeding if full feeding is provided right away. The response cannot be reduced to phosphate and potassium dynamics and plasma concentrations; refeeding also involves less well-known metabolic consequences associated with cardiac, respiratory, and liver dysfunction/failure. Progressive feeding over 3–4 days (so-called restrictive strategy) as realised in the recent trial achieves mortality reduction [31]. After this early refeeding phase, unbalanced regimens with abnormally high proportions of either glucose or fat both lead to de novo lipogenesis [19].

The 2017 ESICM guideline recommends early EN within 48 h [32] based on a meta-analysis of trials showing a significant reduction of infectious complications with this strategy. Early EN may help to preserve gut mucosal integrity and microbiome and also alleviate GI dysfunction/feeding intolerance. At the same time, early EN may be associated with the risk of aspiration, and therefore requires careful monitoring of gastric filling [32] as well as physical precautions to reduce the risk of aspiration, including elevated head-of-bed.

Early parenteral nutrition (PN) allows the provision of macro- and micro-nutrients preventing underfeeding without the potential benefits to the gut. The uncertainties come from the lack of exact determinants of the dose of nutrients required in the acute phase of critical illness. Indeed, enteral feeding intolerance in the early phase of severe illness may possibly be seen as an adaptive process, whereas the use of PN may increase the risk of overfeeding.

## 5. What Do We Know about Early vs. Late Nutrition?

Studies comparing early PN in adults [33] and children [34] have shown impaired outcome with early supplemental PN (SPN) aiming for full equation-based energy targets during the first two days in the intensive care unit (ICU). The hypothesis of PN being the sole cause was not confirmed in the most recent trial where EN and PN were administered at a similar rate [2]. In an Australian study, early PN in patients with contraindications for EN did not increase infectious complications, but was associated with a significant reduction in mechanical ventilation time [35]. Moreover, the Swiss SPN trial applying supplemental PN from day 4 on in patients not achieving targeted energy delivery with EN showed positive effects of SPN vs. prolonged underfeeding [36]. The hypothesis that it is not PN itself but rather full energy delivery in the early phase (more likely achieved by PN as compared to EN) that explains the adverse outcome is further supported by recent meta-analysis [1]. That feeding below actual energy expenditure during the first days in the ICU does not result in worse outcomes compared to provision of full equation-based target is supported by several studies [37,38,39]. However, different substrate compositions and their relative proportions of total energy target used in these trials [38,39] complicate the interpretation of results. Moreover, possible adverse effects of early full nutrition need to be considered. It cannot be excluded that the adverse effect of early full feeding is balanced with an adverse effect of prolonged underfeeding. The TopUp trial, delivering early SPN without testing feeding tolerance in patients with body mass indexes (BMIs) of either ≤25 or ≥35 kg/m^2^ and presenting acute respiratory failure requiring mechanical ventilation, did not observe any significant differences [40]. The previously mentioned PepUp protocol was not successful in all patients [29,30]. It is not clear whether trying to overcome feeding intolerance by all means (prokinetics, increased caloric density, etc.) is beneficial.

Based on existing weak evidence, the initiation of nutrition early via the enteral route (whilst not aiming for coverage of full energy expenditure during the first days after ICU admission) is recommended [32]. There is no evidence supporting early PN in the absence of malnutrition, whereas it could be considered in patient groups with obvious contraindications for EN anticipated to persist for prolonged periods.

Importantly, most of these studies have used different equations to calculate “full energy needs”, whereas all these equations have been shown to lead to overfeeding [41]. The “full” needs of these studies are unlikely to represent actual needs, complicating comparisons and the interpretation of results.

During the last decade, we have learned that:

- Early full nutrition, whatever the route, is harmful and associated with more hyperglycemia, infections, and organ dysfunctions;
- Early EN is beneficial, mainly due to its non-nutritional advantages, and possibly due to its (nearly systematic) slow progression to full feeding;
- Early PN does not have positive enteral effects and incurs larger risks of overfeeding, especially when equations are used to determine the energy target;
- Early low-dose PN may have beneficial outcome effects, particularly in malnourished patients.

Available evidence and open questions are summarized in Table 2.

## 6. Is There a Safe Limit for Underfeeding?

Observational trials conducted with EE measurement by indirect calorimetry [42,43,44] have consistently shown that there is a limited tolerance to the deficit of extrinsic intakes. Increase in malnutrition-related complications starts at −50 kcal/kg body weight (BW) (i.e., about −4000 kcal), and is nearly certain when −100 kcal/kg BW are exceeded [42,43,45] within the first week. Based on these studies, on the physiological considerations of fasting metabolism, and on calorimetry-steered supplemental PN studies [36,46], the limit for a safe tolerance of fasting (absence of nutrition) might be around 3 to 4 days after ICU admission. During underfeeding, cumulative energy deficit seems a useful concept to guide decisions. The latter is calculated as the difference between the measured EE and the sum of provided feeding and non-nutritional energy delivery during the same period.

It is not easy to identify the time point appropriate for advancing to full feeding. While progression over 3–4 days seems beneficial [31], there is no rationale for restricting energy delivery after the first week (criterion used in several studies) in patients entering the recovery phase. It is uncertain whether and for how long this period of extrinsic feeding restriction can or should be maintained in patients staying hemodynamically unstable for more than 72 h, and in those who become chronically critically ill. We know indeed that muscle catabolism results in a massive loss of lean body mass during the first 2 weeks [47], and nutrition therapy may be involved either positively or negatively in its development.

Taken together:

- Some degree of underfeeding in the early phase is probably safe and possibly beneficial;
- Underfeeding beyond 7 days is harmful;
- The limit for safe underfeeding between days 3 and 7 remains unresolved;
- We suggest that a cumulated extrinsic energy deficit between −50 and −100 kcal/kg could help to identify the increase in risk of malnutrition-related complications.

## 7. How to Transfer Our Current Knowledge to Practice?

**Figure 2:**
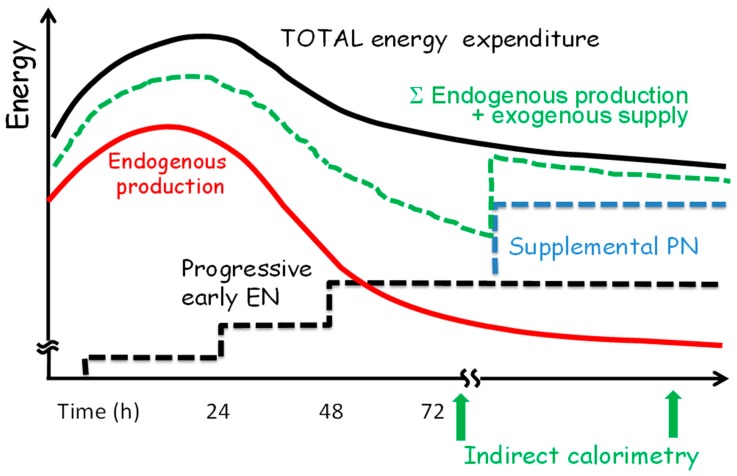
Nutrition strategy to prevent refeeding syndrome and avoid both overfeeding and underfeeding in critical illness (adapted from [14]): dotted black lines represent progressive EN; dotted blue lines represent optional supplemental PN; dotted green lines represent the sum of endogenous energy production and feeding. EN, enteral nutrition; PN, parenteral nutrition.

Based on current knowledge, energy provision should be progressive and EN should be preferred in critically ill patients who are not able to eat. The initiation of nutrition early via enteral route, not aiming at coverage of full measured EE during the first days after ICU admission, is recommended [32]. The strategy for the progression of EN proposed in Figure 2 also applies for early PN when the latter is indicated. The figure shows that in the case of non-progressing EN, combination with PN might be considered.

For metabolic reasons, feeding to cover full measured (or estimated) EE should not be the aim during the acute phase of illness, independent of the route. This statement is supported by the recent analysis of outcome according to the ratio of energy delivery to measured EE [51]. It is noteworthy that there is no data concerning an eventual oral diet restriction in the acute phase. However, critical illness itself limits oral and enteral nutrition with GI dysfunction, anorexia, and feeding intolerance.

Based on the above studies and considerations, we suggest that:

- Early feeding should always be below the actual energy expenditure.
- EN should be started within the first 48 h if an oral diet is not possible, and in the absence of contraindications.
- Full energy requirements should be gradually achieved by day 4 after ICU admission.
- The period of fasting should be limited to a maximum of 3–4 days after ICU admission.
- Intentional underfeeding should not be applied beyond 4 days.
- If EN is not covering 60% of caloric needs by day 4, PN should be considered.
- The effect of extrinsic energy provision significantly below EE for prolonged duration is not clear. Such underfeeding for up to 7 days may be considered in patients without underlying malnutrition.
- Clinicians should strive for determination of EE based either on indirect calorimetry or VCO_2_ measurements [52].

## 8. How to Improve the Knowledge and Patient Management?

Many processes in critically ill patients go beyond normal physiology. Several recent studies were unsuccessful in improving outcome with increased energy and protein delivery. It is possible that there are narrow individual windows for optimal timing, route, and amount of nutrition. The recent data indicate that the issue also includes the composition of feeds, and particularly of the dose of proteins [51]. Adaptive and maladaptive responses of the body need to be better understood and observed, and accordingly supported or suppressed. Therefore, the most important task for future research is to trace the dynamics of the glucose, protein, and lipid metabolism and the development of tools for metabolic monitoring at the bedside. Only precise monitoring tools will allow meaningful research on targeted interventions. Meanwhile, it needs to be acknowledged that different metabolic states may make the same interventions either harmful or beneficial. Studies should focus on a period between days 3 and 7 in the ICU to refine the recommendations on the route, caloric, macro- and micronutrient delivery for this “intermediate” period.

Many domains remain unexplored: e.g., little is known on the best feeding strategy in patients with very high risk of aspiration (e.g., dysphagia combined with gastroparesis, post-esophageal surgery, non-invasive ventilation). Studies exploring metabolic patterns associated with chronic critical illness (i.e., ICU stays longer than 2 weeks) are warranted. Finally, we suggest that future studies should use clear definitions and carefully plan sub-analyses on patient categories.

## 9. Conclusions

Energy expenditure and substrate utilisation are deeply modified by critical illness, whereas bedside markers to assess the endogenous energy production and adapt extrinsic feeding are missing. Early EN, started at a slow rate and increased gradually over 3–4 days, should be considered as a routine feeding approach in ICU patients, due to additional non-nutritional benefits compared to PN. Compared to early full feeding by either route, late feeding may have advantages, but this effect is most likely a dose-effect.

## Figures and Tables

**Table 1:** Definitions of the concepts used in the review.

Term	Definition
Energy	A property of the matter expressed in calories; 1 Calorie (kcal) is the energy needed to increase the temperature of 1 kg of water by 1 °C.
Energy expenditure (EE)	Sum of internal heat produced (endogenous energy production) and external work. The internal heat produced is mainly a sum of basal metabolic rate (BMR) and of the thermic effect of food. It is measured by indirect calorimetry. Importantly, equations used to estimate EE cannot be considered as actual EE.
Energy requirement	Energy from essential nutrients necessary to maintain energy homeostasis. In health, its magnitude depends mainly on age, gender, and physical activity level; in illness, many other factors influence it.
Endogenous energy production	Energy that is produced from internal resources (glucose, proteins, and lipids) that are degraded to produce adenosine triphosphate (ATP). The endogenous glucose production (glycogenolysis and gluconeogenesis) is the first to be activated during fasting to provide it to glucose-dependent organs. In acute illness, endogenous glucose production is increased, but the magnitude and duration of this activation is variable.
Extrinsic energy provision	Includes nutrition and energy-containing medications or fluids administered to patients for non-nutritional purposes.
Energy target	Target for extrinsic energy provision, prescribed by physicians and expressed in kcal/day. Often the target is set to match actual energy expenditure (full target).
Fasting	Complete interruption of feeding: different patterns are possible, such as intermittent fasting, which is now considered a promising weight-loss strategy [4].
Full feeding	Delivery of energy to completely cover equation-estimated target or measured EE: as equations are inexact, only the latter enables a real appreciation of the level of delivery in relation to actual EE.
Nutrient restriction	Reduction of a particular or total nutrient intake without causing malnutrition or biological changes known to shorten animal life span [5]. The reduction can be relative to the subject’s previous intake before intentionally restricting calories, or relative to an average person of similar body type.
Hypocaloric feeding	Feeding below target, or feeding to a target that is deliberately below estimated target or measured EE. Term used to describe feeds with an energy density of ≤1 kcal/mL.
Overfeeding	Feeding quantities of energy that exceed 110% of measured EE.
**Timing of feeding interventions**	
- Early	48 h after intensive care (ICU) admission
- Intermediate	Days 3 to 7 after ICU admission
- Late	Beyond the first week in the ICU

**Table 2:** Evidence and questions regarding early vs. late nutrition in the critically ill (non-exhaustive list).

Concept	Recent Evidence	Open Questions
Nutrition	Essential for survival. Malnutrition causes complications (infections, delayed wound healing).	Is there a phase in critical illness, where nutrition itself might be harmful? Is there a cut-off for energy and substrate dosage?
Timing	“Early” = within 48 h. Rationale: Most of the studies used cut-off of 48 h for “early”. Meta-analyses used either 24 h [48], 48 h [32], or no specified cut-off.	Optimal cut-off for the time-point of early? Optimal time-point for “delayed” in cases “early” was not possible? Optimal progression rate of feeding (EN and PN)
Early vs. delayed EN	Early EN is preferred for mainly non-nutritional reasons. Rationale: Proven benefit of early EN regarding infections in several meta-analyses (low certainty of evidence) [32,49].	Influence of the route of application (gastric vs. jejunal)?
Early EN vs. early PN	Early EN is preferred. Rationale: Proven benefit of early EN regarding infections in one meta-analysis (low certainty of evidence) [32]. One large RCT with similar dosage in EN vs. PN showed no difference in main outcomes [2].	Were the negative effects of early PN observed in studies [1,33] caused solely by dosage and not by route? Did the absent/insufficient glucose control play a role?
Early vs. delayed PN	In patients with contraindications for EN for prolonged time, early PN might be considered. Rationale: One large RCT in patients with relative contraindications to early EN showed shorter MV duration and better strength at 60 days with early PN [35].	In which patients should early PN be considered, at which time point and in which dosage?
Early full vs. early progressive EN	Early EN should be initiated at a rate below actual EE. Rationale: One small RCT showed increased mortality with early full EN with elevated targets (30 kcal/kg) [50]. In several studies, hypocaloric EN during the first week of the ICU stay resulted in similar outcomes [39].	How to determine the optimal extrinsic energy supply in the early phase? At which time-point should the measured EE be fully covered? (Ideally, based on metabolic stage identified with respective future monitoring tool).
Feeding progression (build-up)	Progressive feeding (restricted build-up) should be preferred for both early and late feeding to enable progressive reactivation of metabolism. Rationale: Restricted build-up improved outcome in one RCT in patients developing refeeding syndrome [31]. This is further supported by the above-mentioned INTACT study showing higher mortality with early full feeding [50].	Optimal progression (build-up) of early nutrition? Optimal progression (build-up) of delayed nutrition?

EN, enteral nutrition; PN, parenteral nutrition; RCT, randomized controlled trial; MV, mechanical ventilation; EE energy expenditure, only the most recent studies based on authors’ selection are referenced.
